# Mortality trends and socioeconomic inequalities in sickle cell disease in Colombia, 2012–2023: a population-based study

**DOI:** 10.1186/s13023-026-04475-3

**Published:** 2026-06-30

**Authors:** Uriel Palacios-Barahona, Juan Felipe Combariza

**Affiliations:** 1 Cancer and Hematology Institute, Hospital Universitario Mayor Méderi, Calle 24 No. 29–45, Bogotá, Colombia; 2 Universidad del Rosario, Bogotá, Colombia; 3 Cancer and Hematology Department, Clínica Colsanitas S.A, Bogotá, Colombia; 4 Cancer and Hematology Department, Clínica Universitaria Colombia, Bogotá, Colombia

**Keywords:** Sickle cell disease, Mortality, Temporal trends, Socioeconomic inequalities, Multidimensional poverty, Colombia

## Abstract

**Background:**

Sickle cell disease (SCD) is one of the most common monogenic disorders worldwide and remains associated with substantial mortality in low- and middle-income countries. In Colombia, population-level evidence on long-term mortality trends and socioeconomic inequalities in SCD is limited.

**Methods:**

We conducted an ecological time-series study using death certificate data from the Colombian National Administrative Department of Statistics (DANE). Age-standardized mortality rates (ASMRs) were calculated using the WHO world standard population. Temporal trends were assessed using joinpoint regression and mixed-effects Poisson models. Departments were classified into tertiles of socioeconomic development using the Multidimensional Poverty Index. Additional analyses examined differences by sex and urban–rural residence, and interrupted time-series and excess mortality analyses assessed the COVID-19 period (2020–2021).

**Results:**

A total of 675 SCD-related deaths were recorded between 2012 and 2023. The national ASMR increased from 0.055 per 100,000 in 2012 to 0.143 per 100,000 in 2023. In mixed-effects Poisson models, mortality increased (rate ratio [RR] per year = 1.03; 95% CI: 1.01–1.06; *p* = 0.002). Joinpoint analysis showed a rising trend among males (AAPC = + 8.20%; 95% CI: 0.98 to 15.24; *p* = 0.029). Mortality varied across departments, with the highest rates in Caribbean regions. Departments with low socioeconomic development had higher mortality than those with high development (RR = 3.00; 95% CI: 1.19–7.61; *p* = 0.020), and this association strengthened after adjustment for sex and area of residence (RR = 4.00; 95% CI: 1.60–9.97; *p* = 0.003). During 2020–2021, observed deaths were lower than expected based on pre-pandemic trends (standardized mortality ratio = 0.80; 95% CI: 0.68–0.95).

**Conclusions:**

SCD mortality in Colombia increased between 2012 and 2023, marked by territorial and socioeconomic inequalities. The apparent decline during the COVID-19 period likely reflects under-registration rather than a true reduction in mortality.

**Supplementary Information:**

The online version contains supplementary material available at https://doi.org/10.1186/s13023-026-04475-3.

## Introduction

Sickle cell disease (SCD) is the most common monogenic disorder worldwide and results from a point mutation in the β-globin gene (HBB; Glu6Val) that leads to the production of hemoglobin S (HbS). The disease is characterized by chronic hemolytic anemia, recurrent vaso-occlusive complications, progressive multi-organ damage, and reduced life expectancy [1]. According to the Global Burden of Disease Study, SCD accounted for more than 34,400 deaths worldwide in 2021, with the highest burden concentrated in sub-Saharan Africa, the Caribbean, and selected regions of the Americas [2].

Over recent decades, childhood survival in high-income countries has improved substantially because of the implementation of comprehensive care strategies, including newborn screening, penicillin prophylaxis, vaccination, stroke prevention, hydroxyurea therapy, transfusion programs, and curative approaches such as hematopoietic stem cell transplantation [3–6]. In contrast, mortality remains high in low- and middle-income countries, where delayed diagnosis, limited newborn screening, and restricted access to specialized care continue to compromise outcomes [7]. In Latin America, available evidence from Brazil shows that SCD mortality remains higher than in high-income settings despite advances in screening and care, underscoring persistent regional inequities [8].

Colombia is a middle-income nation with approximately 51 million inhabitants and an ethnically diverse demographic, including an estimated 9.3%Afro-descendantsnt, predominantly situated along the Caribbean and Pacific coastal regions [9]. Previous epidemiological studies have reported elevated HbS trait frequencies in Afro-Colombian communities, particularly in departments such as Chocó, Bolívar, and Valle del Cauca [9, 10]. Although national newborn screening initiatives have expanded in recent years, including the implementation of Resolution 3280 of 2018, population-level evidence on SCD mortality remains limited [11, 12]. In addition, the COVID-19 pandemic may have affected both health-care access and the completeness of death registration, potentially influencing recent mortality estimates [13].

Beyond biological and demographic factors, socioeconomic context may strongly shape SCD outcomes by influencing access to timely diagnosis, disease-modifying treatment, and specialized longitudinal care. In Colombia, the Multidimensional Poverty Index (MPI), reported annually by the National Administrative Department of Statistics (DANE), offers an opportunity to examine whether geographic differences in mortality reflect not only variation in disease burden but also structural inequities in health-system capacity and treatment access [14].

Despite these considerations, no nationwide study has comprehensively evaluated temporal trends and socioeconomic inequalities in SCD mortality in Colombia using standardized epidemiological methods. Therefore, this study analyzed SCD mortality in Colombia from 2012 to 2023 using national death certificate data from DANE. We aimed to characterize temporal trends, assess sex-specific and territorial disparities, examine differences by urban–rural residence, and evaluate socioeconomic inequalities at the departmental level. We also conducted sensitivity analyses to assess the potential impact of the COVID-19 period on observed mortality patterns.

## Materials and methods

### Study design and data sources

We conducted a population-based ecological study of mortality associated with sickle cell disease (SCD) in Colombia between 2012 and 2023. An ecological design was appropriate because mortality outcomes were analyzed at the population level using national death records, and individual-level denominators were not available. The study period began in 2012 to ensure consistency with the availability of standardized mortality data following the restructuring of the Colombian mortality database by the National Administrative Department of Statistics (Departamento Administrativo Nacional de Estadística, DANE).

Mortality data were obtained from individual death certificates compiled by DANE. We included deaths in which SCD was recorded as the underlying cause of death, identified using International Classification of Diseases, 10th Revision (ICD-10) codes D57, D57.1, D57.2, D57.3, D57.4, and D57.8. After excluding seven records with missing department of residence and one record with unspecified age, 675 deaths were included in the primary analysis. Annual population denominators were obtained from official DANE demographic projections. Mortality and population datasets were accessed on June 7, 2025 [15, 16].

### Study variables

The primary analytic variables were year of death (2012–2023), sex (male or female), age (5-year groups), department of residence, and area of residence (urban or rural). Urban areas were defined as municipal seats delimited by the official urban perimeter, whereas rural areas comprised the remaining territory, including populated centers and dispersed rural populations [17].

The primary outcome was the annual age-standardized mortality rate (ASMR) due to SCD, expressed per 100,000 population. The primary temporal analysis consisted of segmented log-linear regression of the national ASMR series to identify changes in mortality trends over time.

### Socioeconomic contextual variable

A contextual socioeconomic variable was constructed using the Multidimensional Poverty Index (MPI), reported annually since 2018 by the Colombian National Administrative Department of Statistics (DANE), based on the National Quality of Life Survey [14]. For each department, we calculated the mean MPI across 2018–2023 to represent structural socioeconomic conditions during the most recent period with comparable data availability. Departments were categorized into tertiles of the national MPI distribution and classified into higher, intermediate, and lower socioeconomic development, with lower MPI values indicating higher socioeconomic development and higher MPI values indicating lower socioeconomic development.

This approach was intended to capture relatively stable territorial deprivation while minimizing temporal collinearity with mortality trends, given the low year-to-year variability of the MPI as a structural contextual indicator. Analyses involving socioeconomic context were restricted to the 2018–2023 period to ensure consistency with the available MPI series (Supplementary Material Table S1).

### Age-standardized mortality rates

Annual mortality rates were calculated for 2012–2023 and age-standardized using the direct method with the World Health Organization (WHO) world standard population [18]. Standardization was performed using 5-year age groups, and rates were expressed per 100,000 inhabitants. ASMRs were estimated overall and stratified by sex, department, and area of residence.

### Temporal trend analysis

Temporal trends in age-standardized mortality rates were assessed using the Joinpoint Regression Program, National Cancer Institute [19]. Log-linear models were fitted to estimate annual percent change (APC) and average annual percent change (AAPC). Statistical significance was evaluated using Monte Carlo permutation tests with 4,499 permutations and a two-sided α level of 0.05. Trends were classified as rising (significant APC > 0), falling (significant APC < 0), stable (non-significant APC between − 0.5% and + 0.5%), or non-significant change (non-significant APC with an absolute value > 0.5%). Stratified analyses were conducted by sex and by area of residence (urban/rural). When joinpoint models were not estimable because of sparse counts or unstable annual rate series, this was explicitly reported.

### Mixed-effects poisson regression

To evaluate temporal trends while accounting for territorial heterogeneity, we fitted a mixed-effects Poisson regression model with a log link using death counts aggregated by department (*d*), year (*t*), and sex (*s*). The model included year (centered at 2017) and sex as fixed effects, a population offset (log [Pop]), and department as a random intercept (*u*_*d*_ ~ N (0, sigma^2^_dept_)). An initial year × sex interaction term did not significantly improve model fit (likelihood ratio test, *p* > 0.05); therefore, the final model reported only main effects. Model selection between Poisson and negative binomial mixed-effects specifications was based on information criteria and diagnostic assessment. The Poisson model showed the best parsimonious fit (AIC = 4,316) and was retained for the main analysis.

The model is specified as follows:

$$\eqalign{& {\rm{log}}\left( {{\rm{E}}\left[ {{\rm{Death}}{{\rm{s}}_{{\rm{dts}}}}} \right]} \right){\rm{ = log}}\left( {{\rm{Po}}{{\rm{p}}_{{\rm{dts}}}}} \right) \cr & \,\,\,\,\,\,\,\,{\rm{ + }}{\beta _{\rm{0}}}{\rm{ + }}{\beta _{\rm{1}}} \cdot {\rm{t + }}{\beta _{\rm{2}}} \cdot {\rm{Sex + }}{{\rm{u}}_{\rm{d}}} \cr} $$

To assess the role of territorial socioeconomic conditions, we fitted separate mixed-effects Poisson regression models restricted to the 2018–2023 period. The first model included centered time, socioeconomic context (MPI tertiles; reference category: high socioeconomic development), the population offset, and department as a random intercept. A second model additionally included area of residence (urban/rural), sex, and a socioeconomic context × area of residence interaction term to evaluate whether the socioeconomic gradient differed between urban and rural populations.

Rate ratios (RRs) and 95% confidence intervals (CIs) were reported. Statistical significance was defined as a two-sided α level of 0.05. Full model coefficients, standard errors, p-values, confidence intervals, goodness-of-fit statistics, and model diagnostics are provided in the (Supplementary Material Table S2-S6).

### Sensitivity analysis: COVID-19 impact

We performed an interrupted time series (ITS) analysis to assess the potential influence of the COVID-19 pandemic on SCD mortality. The ITS model incorporated a binary indicator for the pandemic period (2020–2021), a time-since-pandemic variable, and their interaction. Excess mortality was estimated by comparing observed deaths during 2020–2021 with expected counts derived from the pre-pandemic trend model. The standardized mortality ratio (SMR) with 95% confidence intervals was calculated. Additionally, sensitivity analyses re-estimated the model excluding the pandemic years and restricting to the pre-pandemic period (2012–2019) to assess the robustness of temporal trend estimates (Supplementary Material Table S7).

### Sensitivity analyses

Because ICD-10 code D57.3 corresponds to sickle cell trait rather than sickle cell disease and could therefore introduce misclassification when registered as the underlying cause of death, we performed an additional sensitivity analysis excluding deaths coded as D57.3. Five deaths were excluded, leaving 670 deaths for the sensitivity analysis. The principal temporal analyses, including the mixed-effects Poisson model and joinpoint regression, and the socioeconomic inequality models were re-estimated after exclusion of these records. Results were compared with the primary analysis to assess whether inclusion of D57.3 materially affected the main conclusions (Supplementary Table S8).

### Software

All analyses were performed in R version 4.4.2. Additional implementation details and package-specific outputs are provided in the Supplementary Material S9.

### Ethics statement

This study used publicly available, de-identified secondary mortality and population data from DANE. Because no identifiable individual-level information was accessed and no direct participant contact or intervention occurred, the study was considered exempt from formal ethics review under local regulations.

## Results

### Overall mortality burden

From 2012 to 2023, a total of 675 deaths from sickle cell disease were registered in Colombia. Males accounted for 344 deaths (51.0%), and females accounted for 331 (49.0%). The annual number of deaths increased nearly threefold, from 25 in 2012 to 74 in 2023, with a median of 58 deaths per year. Over the same period, the national age-standardized mortality rate (ASMR) increased from 0.055 per 100,000 population in 2012 to 0.143 per 100,000 population in 2023 (Table 1).

**Table 1:** Summary of national mortality burden from sickle cell disease in Colombia, 2012–2023

Metric	Value
Total deaths (2012–2023)	675
Female deaths	331 (49.0%)
Male deaths	344 (51.0%)
Deaths in 2012	25
Deaths in 2023	74
National ASMR in 2012	0.055 (per 100,000)
National ASMR in 2023	0.143 (per 100,000)

ASMR = age-standardized mortality rate per 100,000 population. Rates were standardized using the WHO world standard population. Multiply rates by 10 to express them per 1,000,000 population for graphical display

### National temporal trends

**Fig. 1:**
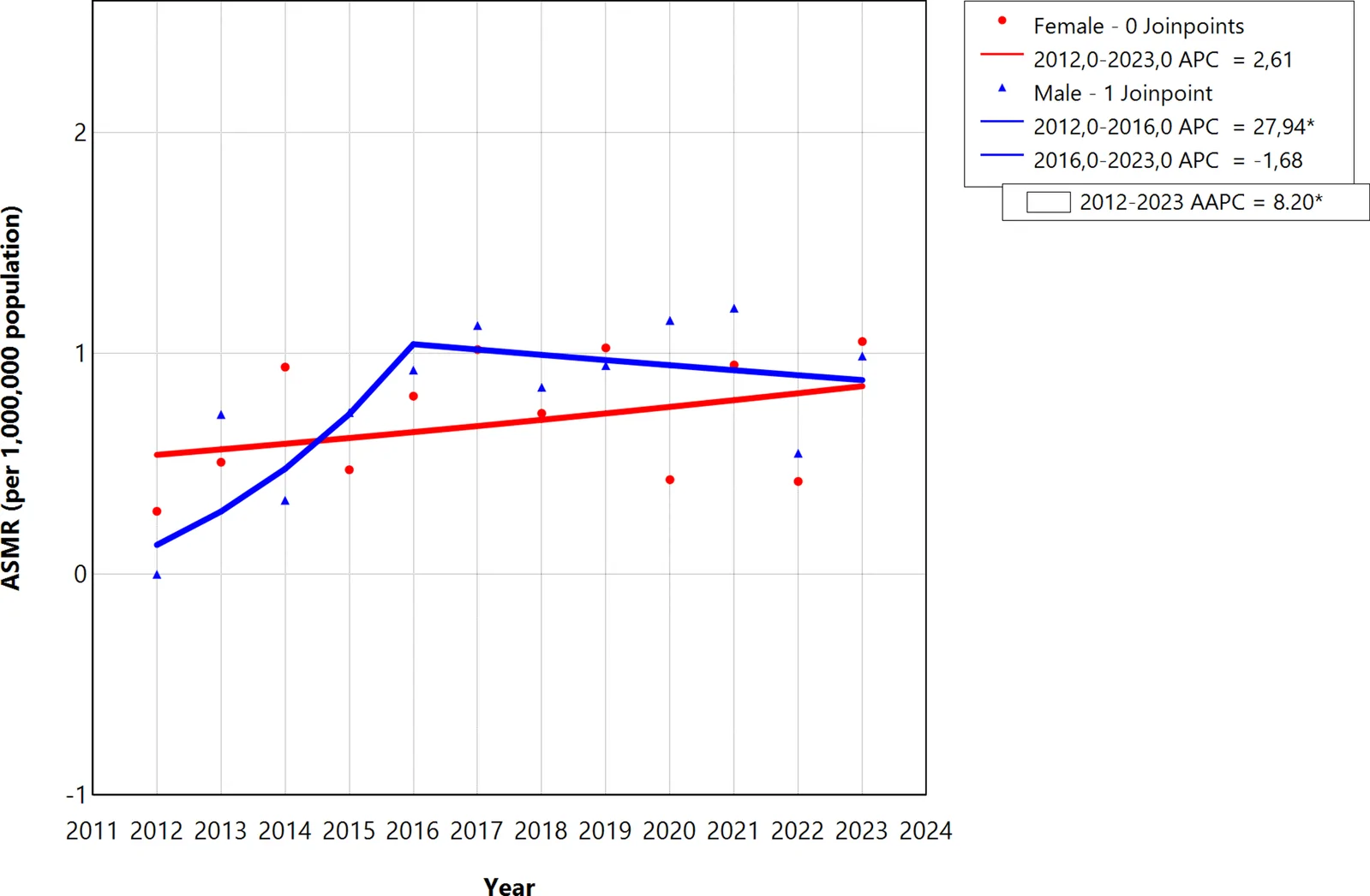
National age-standardized mortality trends in sickle cell disease by sex, Colombia, 2012–2023. For visualization purposes, age-standardized mortality rates in this figure are displayed per 1,000,000 population; all statistical analyses were conducted using rates expressed per 100,000 population

Joinpoint regression showed sex-specific differences in national mortality trends. Among females, no joinpoints were identified, and the overall 2012–2023 trend was classified as a non-significant change (AAPC = + 2.61%; 95% CI: −3.41 to 8.87; *p* = 0.390). Among males, one joinpoint was identified in 2016. Mortality showed a rising trend during 2012–2016 (APC = + 27.94%; 95% CI: 9.65 to 91.01; *p* = 0.004), followed by a non-significant change during 2016–2023 (APC = − 1.68%; 95% CI: −28.45 to 5.60; *p* = 0.532). Across the full study period, the male trend remained rising overall (AAPC = + 8.20%; 95% CI: 0.98 to 15.24; *p* = 0.029) (Fig. 1; Table 2).

These findings were consistent with the mixed-effects Poisson regression model, which estimated a significant 3.0% annual increase in mortality over the full study period after adjustment for sex and department-level heterogeneity (RR per year = 1.03; 95% CI: 1.01–1.06; *p* = 0.002). No significant difference in mortality was observed between males and females after adjustment (RR = 1.08; 95% CI: 0.93–1.25; *p* = 0.327).

**Table 2:** Joinpoint analysis of national age-standardized mortality trends in sickle cell disease by sex, Colombia, 2012–2023

Sex	Segment / period	APC or AAPC (%)	95% CI	*p*-value	Trend classification
Female	2012–2023 (AAPC)	+ 2.61	−3.41 to 8.87	0.390	Non-significant change
Male	2012–2016 (APC)	+ 27.94	9.65 to 91.01	0.004	Rising
Male	2016–2023 (APC)	−1.68	−28.45 to 5.60	0.532	Non-significant change
Male	2012–2023 (AAPC)	+ 8.20	0.98 to 15.24	0.029	Rising

### Geographic distribution

Substantial geographic heterogeneity was observed across Colombian departments. The highest average annual mortality rates were concentrated in the Caribbean region, particularly in Bolívar (0.386 per 100,000), La Guajira (0.341), Magdalena (0.334), Cesar (0.296), and Atlántico (0.266). In contrast, Bogotá, D.C. had a substantially lower rate (0.029 per 100,000) despite 26 recorded deaths. Among the ten departments with the highest mortality burden, the ratio between the highest and lowest average annual mortality rates was 13.3:1.0.

### Temporal trends by area of residence

**Fig. 2:**
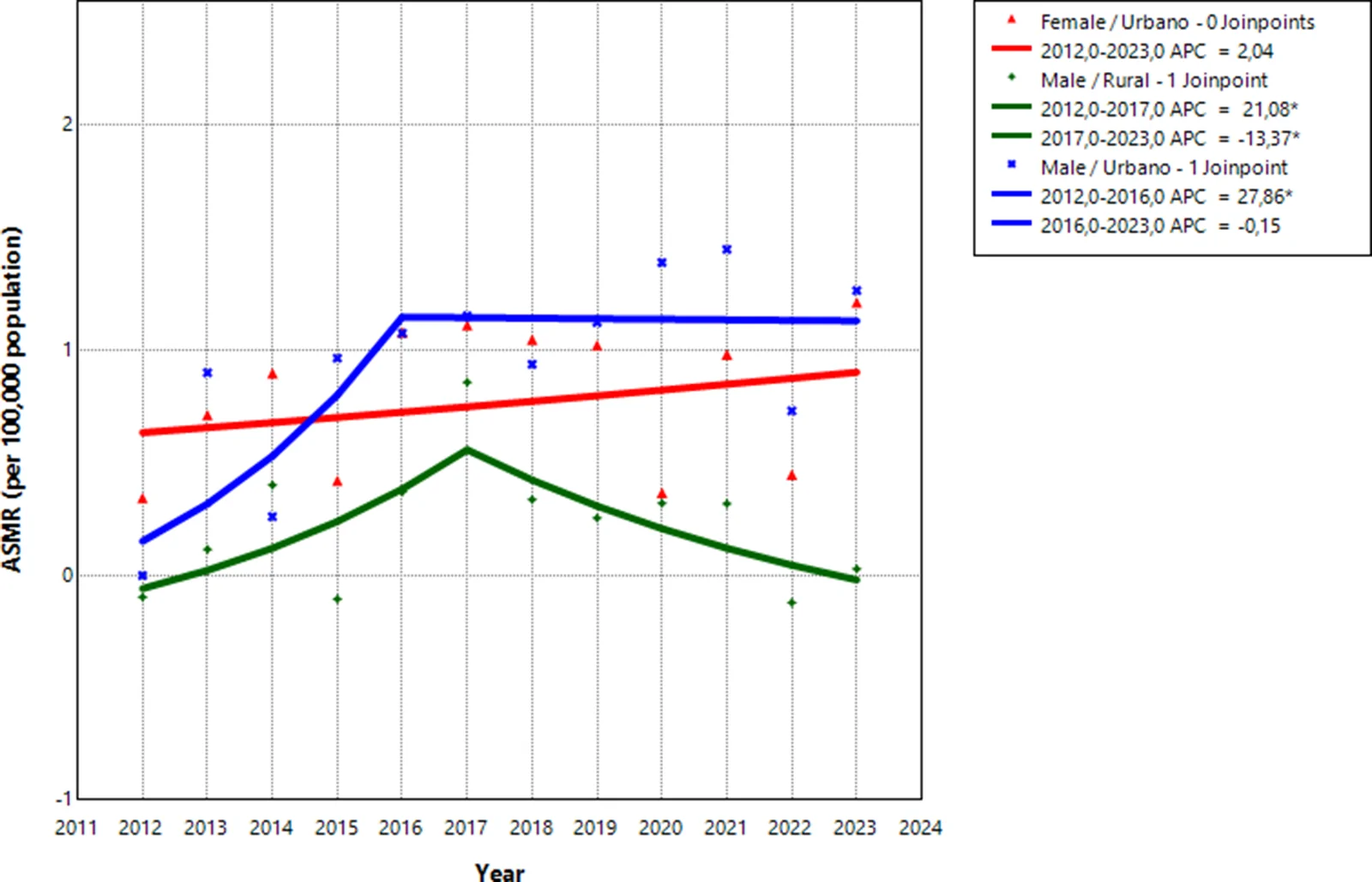
Temporal trends in age-standardized sickle cell disease mortality by sex and area of residence in Colombia, 2012–2023. Joinpoint models for rural females were not estimable because of sparse annual counts and unstable rate series

Stratified analyses by area of residence showed additional heterogeneity. Among urban females, the overall 2012–2023 trend was classified as a non-significant change (AAPC = + 2.04%; 95% CI: −2.80 to 7.22; *p* = 0.442). Joinpoint models for rural females were not estimable because of sparse annual counts and unstable rate series. Among rural males, mortality showed a rising trend during 2012–2017 (APC = + 21.08%; 95% CI: 4.61 to 88.22; *p* = 0.006), followed by a falling trend during 2017–2023 (APC = − 13.37%; 95% CI: −42.16 to − 3.23; *p* = 0.008); however, the overall 2012–2023 trend was classified as a non-significant change (AAPC = + 0.87%; 95% CI: −7.02 to 9.22; *p* = 0.820). Among urban males, mortality increased during 2012–2016 (APC = + 27.86%; 95% CI: 5.71 to 104.78; *p* = 0.015), followed by a stable trend during 2016–2023 (APC = − 0.15%; 95% CI: −32.74 to 11.08; *p* = 0.758), while the overall 2012–2023 trend remained rising (AAPC = + 9.24%; 95% CI: 0.97 to 17.59; *p* = 0.032) (Fig. 2) (Supplementary Table S10).

### Socioeconomic inequalities in mortality (2018–2023)

Between 2018 and 2023, 379 deaths from sickle cell disease were recorded. Although deaths were numerically distributed across departments with high (35.4%), intermediate (33.5%), and low (31.1%) socioeconomic development, mortality rates were consistently higher in low-development departments across all years. In the primary mixed-effects Poisson model, departments with low socioeconomic development had a three-fold higher mortality rate than those with high development (RR = 3.00; 95% CI: 1.19–7.61; *p* = 0.020), whereas the difference for intermediate-development departments was not statistically significant (RR = 1.60; 95% CI: 0.61–4.15; *p* = 0.340). No significant temporal trend was observed within the 2018–2023 period (RR = 1.00; 95% CI: 0.94–1.06; *p* = 0.980) (Table 3) (Supplementary Figure S1-S3).

**Table 3:** Poisson mixed-effects regression with socioeconomic stratification, sickle cell disease mortality, Colombia, 2018–2023

Parameter	Estimate	SE	RR	95% CI Lower	95% CI Upper	*p*-value
Intercept	-14.557	0.357	—	—	—	< 0.001
Time (centered)	-0.00	0.03	1.00	0.94	1.06	0.980
SES: Intermediate vs. High	0.47	0.49	1.60	0.61	4.15	0.340
SES: Low vs. High	1.10	0.47	3.00	1.19	7.61	0.020

Socioeconomic development level based on MPI tertiles; RR, rate ratio; SE, standard error. Reference category: High socioeconomic development. Random effect: department (variance = 0.874, SD = 0.935). ICC = 6.1%. AIC = 2,393

In the model additionally adjusted for sex and area of residence, the excess mortality associated with low socioeconomic development became more pronounced (RR = 4.00; 95% CI: 1.60–9.97; *p* = 0.003). Rural residence was associated with a non-significant tendency toward lower mortality (RR = 0.61; 95% CI: 0.35–1.05; *p* = 0.070), and males showed a non-significant tendency toward higher mortality (RR = 1.20; 95% CI: 0.98–1.46; *p* = 0.080). The socioeconomic context × residence interaction terms were not statistically significant, indicating no statistically significant evidence that the socioeconomic gradient differed by area of residence (Supplementary Table S6).

### Sensitivity analyses: COVID-19 impact

The interrupted time-series analysis did not detect a significant immediate level change during the pandemic period (RR = 0.81; 95% CI: 0.59–1.11; *p* = 0.182), although the post-pandemic slope was lower than the pre-pandemic trend (RR = 0.85; 95% CI: 0.75–0.97; *p* = 0.019). Excess mortality analysis showed that deaths during 2020–2021 were 19.8% lower than expected based on pre-pandemic trends (136 observed vs. 169 expected; SMR = 0.80; 95% CI: 0.68–0.95). Sensitivity analyses confirmed that mortality increased more steeply before the pandemic and remained significantly increasing after excluding 2020–2021, suggesting that the pandemic attenuated but did not reverse the long-term upward trend (Supplementary Table 7).

In the sensitivity analysis excluding five deaths coded as sickle cell trait (ICD-10 D57.3), 670 deaths remained for analysis. Exclusion of these records did not materially alter the principal findings. The increasing temporal trend was retained in the mixed-effects Poisson model (primary analysis: RR per year = 1.03; 95% CI: 1.01–1.06; sensitivity analysis: RR per year = 1.034; 95% CI: 1.012–1.057). The rising overall male trend identified through joinpoint regression was unchanged (AAPC = + 8.20%; 95% CI: 0.98–15.24). Similarly, the socioeconomic gradient remained evident in the primary model (low vs. high socioeconomic development: RR = 3.00; 95% CI: 1.19–7.61) and in the model including sex, area of residence, and their interaction with socioeconomic context (low vs. high socioeconomic development among urban residents: RR = 4.00; 95% CI: 1.60–9.97) (Supplementary Table S9).

## Discussion

This nationwide population-based study provides, to our knowledge, the first comprehensive assessment of temporal trends and socioeconomic inequalities in sickle cell disease (SCD) mortality in Colombia. Four main findings emerge. First, SCD mortality increased over the study period, with the national age-standardized mortality rate rising from 0.055 per 100,000 population in 2012 to 0.143 per 100,000 population in 2023, and the mixed-effects Poisson model indicating a significant annual increase (RR per year = 1.03; 95% CI: 1.01–1.06; *p* = 0.002). Second, marked geographic heterogeneity was observed, with mortality concentrated in Caribbean departments and a 13.3-fold difference between the highest- and lowest-rate departments among the ten departments with the greatest mortality burden. Third, mortality showed a significant increasing trend among males overall (AAPC = + 8.20%; 95% CI: 0.98 to 15.24; *p* = 0.029) and among urban males (AAPC = + 9.24%; 95% CI: 0.97 to 17.59; *p* = 0.032), whereas female trends were not statistically significant. Fourth, departments with low socioeconomic development showed higher population-level documented mortality than high-development departments.

The observed increase in documented SCD-related mortality should be interpreted cautiously in the context of evolving diagnostic and registration practices in Colombia. The early increase, particularly before the male joinpoint in 2016, may partly reflect improved case ascertainment and coding consistency following progressive strengthening of mortality registration and surveillance systems [20]. In earlier years, and particularly in settings with limited diagnostic capacity, deaths occurring among individuals with SCD may have been coded under proximate complications such as infection, acute chest syndrome, stroke, or organ failure rather than under SCD itself. Therefore, the observed upward trend cannot be interpreted solely as evidence of worsening mortality among people living with SCD. Rather, it may reflect a combination of improved recognition and registration of SCD-related deaths and a persistent unmet mortality burden. Expansion of newborn and early-life screening initiatives, including local programs such as PREGEN in Bogotá and the broader national framework introduced under Resolution 3280 of 2018, may also have increased recognition of SCD within the health system [11]. Yet greater visibility in administrative data does not necessarily imply improved survival when access to hydroxyurea, prophylactic care, and specialist follow-up remains uneven [12, 21]. In high-burden Afro-Colombian communities such as San Basilio de Palenque, substantial underuse of hydroxyurea has been documented, underscoring the gap between diagnosis and effective longitudinal management [22].

The socioeconomic gradient is one of the most important findings of this study, but it must be interpreted considering the unequal geographic distribution of SCD in Colombia. Departments with low socioeconomic development had substantially higher population-level documented mortality than those with high development in both the primary model (RR = 3.00; 95% CI: 1.19–7.61) and the model additionally adjusted for sex and area of residence (RR = 4.00; 95% CI: 1.60–9.97). However, departments with greater structural deprivation frequently overlap with territories that have larger Afro-Colombian populations and may therefore also have a higher underlying prevalence of SCD [9, 10]. Because our analyses used the general population as the denominator and reliable department-level prevalence estimates of SCD were unavailable, we cannot distinguish whether the observed gradient reflects a greater number of individuals living with SCD, poorer survival among affected individuals, barriers to comprehensive care, differential registration, or a combination of these mechanisms. Accordingly, these findings should not be interpreted as demonstrating an independent causal effect of poverty on individual mortality. Rather, they identify territories where higher expected disease burden and structural disadvantage converge, creating an intersectional vulnerability that warrants regionally tailored interventions. The absence of a significant socioeconomic context × residence interaction further suggests that this vulnerability is not restricted to rural settings, but may also operate within underserved urban populations [21].

Our findings are broadly consistent with evidence from other Latin American settings, particularly Brazil, where SCD remains associated with substantial regional and social inequalities despite advances in newborn screening [23]. In contrast, high-income countries in North America and Western Europe have reduced early-life mortality through universal newborn screening, penicillin prophylaxis, vaccination, transcranial Doppler surveillance, and earlier access to hydroxyurea [24–26]. National cohort studies from England and France also report low childhood mortality when comprehensive care programs are implemented from birth [3, 27]. However, adult mortality remains a continuing challenge even in resource-rich settings [24], underscoring that the benefits of comprehensive SCD care must extend across the life course.

Our findings align with trends observed in other Latin American nations, notably Brazil, where SCA remains a significant cause of death, characterized by marked regional and socioeconomic inequities. This contrasts sharply with high-income countries in North America and Western Europe, where universal newborn screening, penicillin prophylaxis, and early hydroxyurea initiation have stabilized or reduced pediatric mortality. However, even in these resource-rich settings, adult mortality remains a challenge, emphasizing that the benefits of comprehensive care must extend across the lifespan.

However, improvements in high-income countries are mainly confined to pediatric populations, and adult mortality remains a challenge, emphasizing that the benefits of comprehensive care must extend across the life span. This distinction is relevant because our analysis includes all ages and reflects a context in which comprehensive SCD care is not universally available, emphasizing the importance of screening linked to guaranteed multidisciplinary follow-up.

The sex-specific findings also merit consideration. Although the adjusted national model did not identify a statistically significant overall difference between males and females, joinpoint analyses suggested a more unfavorable temporal pattern among males, particularly urban males. This may reflect a combination of better diagnostic recognition in metropolitan settings and sex-related differences in disease severity, health-care utilization, treatment adherence, or chronic organ complications described in other SCD populations [28]. By contrast, the absence of significant trends in smaller strata especially rural females should be interpreted cautiously given sparse counts and unstable annual rates [29].

The lower-than-expected number of documented SCD-related deaths during 2020–2021 should be interpreted as a pandemic paradox. Health-system disruption, altered access to care, and changes in cause-of-death certification may have contributed to under-ascertainment or misclassification during this period [30–32]. However, this may not be the only explanation. Public health measures implemented during the COVID-19 pandemic, including masking, physical distancing, reduced mobility, and school closures, were associated with marked reductions in the circulation of respiratory pathogens such as influenza [33]. Because respiratory infections are recognized triggers of acute complications in SCD, reduced exposure to infectious pathogens may also have contributed to a temporary reduction in documented SCD-related mortality. These mechanisms are not mutually exclusive, and the present study cannot quantify their relative contribution.

These findings have direct public health implications. Rather than attributing excess mortality solely to socioeconomic deprivation, our findings identify Colombian territories in which a potentially greater underlying SCD burden intersects with structural disadvantage and limited access to comprehensive care. SCD strategies should therefore prioritize high-burden Caribbean departments and other socioeconomically disadvantaged territories through strengthened screening and confirmatory diagnosis, guaranteed access to prophylaxis and hydroxyurea, long-term multidisciplinary follow-up, and improved disease-specific surveillance. Decentralized care pathways, telehealth-supported specialist networks, community health worker models, and stronger referral systems may be especially relevant for historically underserved Afro-descendant populations and other high-burden regions [21, 22, 29].

This study has several limitations. First, as an ecological analysis based on death certificate data, it is vulnerable to underreporting and misclassification, particularly when SCD-related deaths are coded under proximate complications rather than the underlying disease. Temporal improvements in disease recognition and cause-of-death ascertainment after 2012 may also have contributed to the observed increase in documented mortality. Second, reliable department-level prevalence estimates of SCD were unavailable; therefore, mortality rates were calculated using general population denominators, and the models could not adjust for the regional distribution of people living with SCD. Consequently, the observed geographic and socioeconomic gradients may reflect regional differences in disease prevalence, survival, access to care, registration practices, or a combination of these mechanisms, and should not be interpreted as individual-level causal effects of poverty. Third, five deaths in the primary registry-based analysis were coded as sickle cell trait (ICD-10 D57.3). Because this code does not represent SCD, we conducted a sensitivity analysis excluding these records; the principal temporal and socioeconomic findings were materially unchanged. Fourth, the socioeconomic analysis was restricted to 2018–2023 and relied on department-level MPI tertiles, precluding individual-level inference and potentially masking within-department heterogeneity. Finally, some stratified joinpoint models were unstable or non-estimable because of sparse counts, an expected challenge in rare disease mortality analyses. Future research should prioritize linkage studies and prospective individual-level cohorts integrating clinical registries, newborn screening, treatment access, regional prevalence estimates, and mortality outcomes.

## Conclusions

Sickle cell disease-related mortality documented in Colombia increased between 2012 and 2023, with marked territorial and socioeconomic disparities. These disparities identify regions where a potentially higher underlying SCD burden overlaps with structural deprivation and reduced access to comprehensive care, particularly in Caribbean departments. The increasing temporal trend was most evident among males, particularly urban males. The lower documented mortality during the COVID-19 period may reflect disruptions in surveillance and cause-of-death registration, reduced exposure to respiratory infectious triggers during public health restrictions, or both.

Reducing avoidable mortality will require improved disease-specific surveillance, reliable estimates of regional SCD prevalence, and regionally tailored strategies linking screening and confirmatory diagnosis to disease-modifying therapy, longitudinal multidisciplinary follow-up, and decentralized comprehensive care in high-burden territories.

## Supplementary Information

Below is the link to the electronic supplementary material.

Supplementary Material 1

## Data Availability

No datasets were generated or analysed during the current study.

## Abbreviations

APC: Annual percent change
AAPC: Average annual percent change
ASMR: Age-standardized mortality rates
DANE: National Administrative Department of Statistics of Colombia
RR: Rate ratio
SCD: Sickle cell disease
