# Supplementary material for: Mortality trends and socioeconomic inequalities in sickle cell disease in Colombia, 2012–2023: a population-based study
Source: Orphanet J Rare Dis. 2026 Jun 30;21:325. doi: 10.1186/s13023-026-04475-3 (PMC13587389; doi:10.1186/s13023-026-04475-3)
Supplement: Supplementary file 1 [file 13023_2026_4475_MOESM1_ESM.docx]

**Supplementary Material**

**Mortality trends and socioeconomic inequalities in sickle cell disease in Colombia, 2012–2023: a population-based study**

**S1 MPI classification. Socioeconomic contextual variable: Multidimensional Poverty Index tertile classification**

Departments were classified into three categories according to the **mean departmental Multidimensional Poverty Index (MPI)** during **2018–2023**. Tertiles were defined using the national distribution of departmental MPI values as follows:

- **High socioeconomic development (lowest MPI tertile):** departments with the lowest levels of multidimensional poverty and structural deprivation.
- **Intermediate socioeconomic development (middle MPI tertile):** departments in the middle tertile of the national MPI distribution.
- **Low socioeconomic development (highest MPI tertile):** departments with the highest levels of multidimensional poverty and structural deprivation.

The MPI is reported annually by the **National Administrative Department of Statistics (DANE)** and captures multiple dimensions of deprivation, including education, childhood and youth conditions, employment, health, and housing. Higher MPI values indicate greater multidimensional poverty. Because MPI is a structural contextual indicator with limited year-to-year variability at the departmental level, the **2018–2023 period mean** was used in the main analyses to reduce temporal collinearity with annual mortality trends and to represent underlying territorial socioeconomic conditions more robustly.

**Supplementary Table S1. Classification of Colombian departments according to socioeconomic development based on the mean Multidimensional Poverty Index (MPI), 2018–2023.**

| **Department** | **Socioeconomic development category** |
| --- | --- |
| Antioquia | High |
| San Andrés | High |
| Bogotá | High |
| Boyacá | High |
| Caldas | High |
| Cundinamarca | High |
| Quindio | High |
| Risaralda | High |
| Santander | High |
| Valle del Cauca | High |
| Atlántico | Intermediate |
| Bolívar | Intermediate |
| Caquetá | Intermediate |
| Casanare | Intermediate |
| Cauca | Intermediate |
| Huila | Intermediate |
| Meta | Intermediate |
| Norte de Santander | Intermediate |
| Putumayo | Intermediate |
| Tolima | Intermediate |
| Amazonas | Low |
| Arauca | Low |
| Córdoba | Low |
| Cesar | Low |
| Chocó | Low |
| Guainía | Low |
| Guaviare | Low |
| La Guajira | Low |
| Magdalena | Low |
| Nariño | Low |
| Sucre | Low |
| Vaupés | Low |
| Vichada | Low |

Abbreviation: MPI, Multidimensional Poverty Index.

Note: Departments were classified using the mean departmental MPI for 2018–2023 and grouped into tertiles of the national distribution. High socioeconomic development = lowest MPI tertile; Intermediate = middle tertile; Low socioeconomic development = highest MPI tertile. Higher MPI values indicate greater multidimensional poverty.

**Supplementary Table S2. Age-standardized mortality rates (per 100,000 population) from sickle cell disease by sex in Colombia, 2012–2023.**

| **Year** | **Cases Female** | **Cases Male** | **Cases Total** | **ASMR Female** | **ASMR Male** | **ASMR Total** | **Crude Rate Female** |
| --- | --- | --- | --- | --- | --- | --- | --- |
| 2012 | 16 | 9 | 25 | 0.069 | 0.041 | 0.055 | 0.056 |
| 2013 | 21 | 25 | 46 | 0.091 | 0.113 | 0.102 | 0.101 |
| 2014 | 32 | 17 | 49 | 0.135 | 0.074 | 0.104 | 0.107 |
| 2015 | 21 | 26 | 47 | 0.088 | 0.114 | 0.101 | 0.101 |
| 2016 | 29 | 30 | 59 | 0.121 | 0.133 | 0.127 | 0.126 |
| 2017 | 34 | 36 | 70 | 0.142 | 0.153 | 0.148 | 0.148 |
| 2018 | 27 | 29 | 56 | 0.114 | 0.125 | 0.120 | 0.116 |
| 2019 | 35 | 33 | 68 | 0.143 | 0.135 | 0.139 | 0.138 |
| 2020 | 22 | 39 | 61 | 0.084 | 0.156 | 0.120 | 0.121 |
| 2021 | 35 | 40 | 75 | 0.136 | 0.161 | 0.148 | 0.147 |
| 2022 | 21 | 24 | 45 | 0.083 | 0.096 | 0.089 | 0.087 |
| 2023 | 38 | 36 | 74 | 0.146 | 0.140 | 0.143 | 0.142 |

*ASMR = age-standardized mortality rate per 100,000 population, multiply by 10 to convert to rates per 1,000,000. Rates were standardized using the WHO world standard population.*

# Supplementary Table S3: Poisson vs. Negative Binomial specifications for the socioeconomic analysis (2018–2023)

| **Model** | **AIC** | **BIC** | **LogLik** | **Delta AIC** | **Best** |
| --- | --- | --- | --- | --- | --- |
| Poisson | 2,392.6 | 2,427.7 | -1,191.3 | 0 | *** |
| NB (without interaction) | 2,393.2 | 2,435.4 | -1,190.6 | 0.59 |  |
| NB (with interaction) | 2,395.1 | 2,451.3 | -1,189.5 | 2.45 |  |

*AIC: Akaike Information Criterion; BIC: Bayesian Information Criterion; NB: negative binomial. The Poisson model provided the best fit and was selected as the final model.*

# Supplementary Table S4. Complete Poisson mixed-effects model output for temporal trends (2012–2023)

Model specification: Generalized linear mixed model (Poisson family, log link)

Formula: cases ~ time_centered + sex_factor + offset(log_population) + (1 | department_factor)

| **Parameter** | **Estimate** | **SE** | **z-value** | **p-value** |
| --- | --- | --- | --- | --- |
| Intercept | -14.269 | 0.240 | -59.55 | <0.001 |
| Time (centered) | 0.034 | 0.011 | 3.04 | 0.002 |
| Sex (male) | 0.075 | 0.076 | 0.98 | 0.327 |

Random effects: Department (variance = 1.45, SD = 1.20). Number of observations: 16,632; groups: 33 departments. AIC = 4,316; BIC = 4,347.

# Supplementary Table S5. Complete Poisson mixed-effects model output for socioeconomic analysis (2018–2023) — General model

Model specification: Poisson GLMM with socioeconomic context

Formula: cases ~ time_centered + contexto_socioeconomico + offset(log_population) + (1 | department_factor)

| **Parameter** | **Estimate** | **SE** | **RR** | **95% CI Lower** | **95% CI Upper** | **p-value** |
| --- | --- | --- | --- | --- | --- | --- |
| Intercept | -14.557 | 0.357 | — | — | — | <0.001 |
| Time (centered) | -0.001 | 0.030 | 0.999 | 0.942 | 1.060 | 0.98 |
| SES: Intermediate vs High | 0.468 | 0.488 | 1.596 | 0.613 | 4.154 | 0.34 |
| SES: Low vs High | 1.100 | 0.474 | 3.004 | 1.186 | 7.607 | 0.02 |

Random effects: Department (variance = 0.874, SD = 0.935). ICC = 6.1%. R² marginal = 0.015; R² conditional = 0.075. Number of observations: 8,316; groups: 33 departments. AIC = 2,393.

# Supplementary Table S6. Complete Poisson mixed-effects model output for socioeconomic analysis (2018–2023) — Model with area of residence and sex

Model specification: Poisson GLMM with socioeconomic context × residence interaction

Formula: cases ~ time_centered + contexto_socioeconomico * residence + sex + offset(log_population) + (1 | department)

| **Parameter** | **Estimate** | **SE** | **RR** | **95% CI Lower** | **95% CI Upper** | **p-value** |
| --- | --- | --- | --- | --- | --- | --- |
| Intercept | -14.552 | 0.353 | — | — | — | <0.001 |
| Time (centered) | -0.001 | 0.030 | 0.999 | 0.942 | 1.060 | 0.98 |
| SES: Intermediate vs High | 0.613 | 0.479 | 1.845 | 0.722 | 4.716 | 0.20 |
| SES: Low vs High | 1.385 | 0.467 | 3.995 | 1.601 | 9.971 | 0.003 |
| Residence: Rural vs Urban | -0.497 | 0.278 | 0.608 | 0.353 | 1.049 | 0.07 |
| Sex: Male vs Female | 0.178 | 0.103 | 1.195 | 0.976 | 1.462 | 0.08 |
| SES Intermediate × Rural | -0.518 | 0.413 | 0.595 | 0.265 | 1.338 | 0.21 |
| SES Low × Rural | -0.592 | 0.363 | 0.553 | 0.272 | 1.126 | 0.10 |

Random effects: Department (variance = 0.816, SD = 0.903). ICC = 5.7%. R² marginal = 0.030; R² conditional = 0.085. Number of observations: 16,632; groups: 33 departments. AIC = 2,660.

# Supplementary Table S7. Sensitivity analysis: comparison of temporal trend estimates across model specifications

We performed an interrupted time series (ITS) analysis to assess the potential influence of the COVID-19 pandemic on SCD mortality. The ITS model incorporated a binary indicator for the pandemic period (2020–2021), a time-since-pandemic variable, and their interaction. Excess mortality was estimated by comparing observed deaths during 2020–2021 with expected counts derived from the pre-pandemic trend model.

| **Parameter** | **Estimate** | **SE** | **RR** | **95% CI Lower** | **95% CI Upper** | **p-value** |
| --- | --- | --- | --- | --- | --- | --- |
| Intercept | -14.184 | 0.247 | — | -14.668 | -13.701 | <0.001 |
| Time (centered) | 0.071 | 0.021 | 1.074 | 1.029 | 1.119 | 0.001 |
| Sex: Male vs Female | 0.075 | 0.077 | 1.078 | 0.927 | 1.253 | 0.331 |
| Pandemic Period | -0.215 | 0.161 | 0.806 | 0.588 | 1.106 | 0.182 |
| Time Since Pandemic | -0.158 | 0.067 | 0.854 | 0.748 | 0.974 | 0.019 |
| Pandemic Period × Time Since Pandemic | 0.278 | 0.180 | 1.321 | 0.928 | 1.880 | 0.122 |

*RR: rate ratio per year. All models: Poisson mixed-effects regression with log population offset and department random intercept.*

**Supplementary Table S8. Comparison of Mortality Outcomes: Primary and Sensitivity Analysis.**

| **Outcome** | **Primary analysis including D57.3** | **Sensitivity analysis excluding D57.3** | **Interpretation** |
| --- | --- | --- | --- |
| Total deaths, n | 675 | 670 | Five D57.3-coded deaths excluded |
| Temporal trend, RR per year | 1.03 (1.01–1.06); p = 0.002 | 1.034 (1.012–1.057); p = 0.0026 | Significant increasing trend retained |
| Male vs female, RR | 1.08 (0.93–1.25); p = 0.327 | 1.078 (0.927–1.253); p = 0.332 | No statistically significant sex difference retained |
| Female overall trend, AAPC | +2.61% (−3.41 to 8.87); p = 0.390 | +2.61% (−3.41 to 8.87); p = 0.390 | Non-significant change retained |
| Male overall trend, AAPC | +8.20% (0.98–15.24); p = 0.029 | +8.20% (0.98–15.24); p = 0.029 | Rising trend retained |
| Urban male overall trend, AAPC | +9.24% (0.97–17.59); p = 0.032 | +9.24% (0.97–17.59); p = 0.032 | Rising trend retained |
| Low vs high socioeconomic development, primary model, RR | 3.00 (1.19–7.61); p = 0.020 | 3.00 (1.19–7.61); p = 0.020 | Socioeconomic gradient retained |
| Low vs high socioeconomic development among urban residents, interaction model, RR | 4.00 (1.60–9.97); p = 0.003 | 4.00 (1.60–9.97); p = 0.003 | Socioeconomic gradient retained |
| Low SES × rural residence interaction, RR | 0.55 (0.27–1.13); p = 0.100 | 0.55 (0.27–1.13); p = 0.102 | No evidence of effect modification by residence retained |

*Abbreviations: AAPC, average annual percent change; RR, rate ratio; SES, socioeconomic development category based on Multidimensional Poverty Index tertiles. The sensitivity analysis excluded deaths coded as ICD-10 D57.3 because this code corresponds to sickle cell trait rather than sickle cell disease.*

**Supplementary Table S9. Statistical software and packages used in the analysis**

| **Analysis component** | **Software or package** | **Version** | **Purpose** |
| --- | --- | --- | --- |
| General statistical analyses | R | 4.4.2 | Data management, descriptive analyses, model fitting, diagnostic assessment, estimation of contrasts, and calculation of age-standardized mortality rates |
| Joinpoint regression | Joinpoint Regression Program, Statistical Methodology and Applications Branch, Surveillance Research Program, National Cancer Institute, Bethesda, MD, USA | 5.0.5 | Estimation of annual percent change (APC) and average annual percent change (AAPC) |
| Mixed-effects count models | glmmTMB | 1.1.11 | Fitting mixed-effects Poisson and negative binomial count models |
| Model diagnostics | DHARMa | 0.4.7 | Assessment of residual diagnostics and model fit |
| Model performance assessment | performance | 0.15.1 | Evaluation of model performance and diagnostic indicators |
| Estimated marginal means and contrasts | emmeans | 1.11.2-8 | Estimation of marginal means and pairwise or model-based contrasts |

**Supplementary Table S10. Joinpoint analysis of age-standardized mortality trends in sickle cell disease by sex and area of residence, Colombia, 2012–2023**

| **Sex / Residence** | **Segment / period** | **APC or AAPC (%)** | **95% CI** | **p-value** | **Trend classification** |
| --- | --- | --- | --- | --- | --- |
| Female / Urban | 2012–2023 (AAPC) | +2.04 | −2.80 to 7.22 | 0.442 | Non-significant change |
| Female / Rural | 2012–2023 | Not estimable | — | — | — |
| Male / Rural | 2012–2017 (APC) | +21.08 | 4.61 to 88.22 | 0.006 | Rising |
| Male / Rural | 2017–2023 (APC) | −13.37 | −42.16 to −3.23 | 0.008 | Falling |
| Male / Rural | 2012–2023 (AAPC) | +0.87 | −7.02 to 9.22 | 0.820 | Non-significant change |
| Male / Urban | 2012–2016 (APC) | +27.86 | 5.71 to 104.78 | 0.015 | Rising |
| Male / Urban | 2016–2023 (APC) | −0.15 | −32.74 to 11.08 | 0.758 | Stable |
| Male / Urban | 2012–2023 (AAPC) | +9.24 | 0.97 to 17.59 | 0.032 | Rising |

*Joinpoint models for rural females were not estimable because of sparse annual counts and unstable rate series.*

# Supplementary Figure S1. Mortality trends by socioeconomic context, Colombia, 2018–2023

*Description: Line graph showing annual crude mortality rates from sickle cell anemia by socioeconomic context (high, intermediate, and low development) during 2018–2023. Departments with low socioeconomic development consistently show higher mortality rates across all years.*

# Supplementary Figure S2. Forest plot of model results, socioeconomic analysis

*Description: Forest plot displaying rate ratios and 95% confidence intervals from the Poisson mixed-effects regression model with socioeconomic stratification.*

# Supplementary Figure S3. Forest plot of area-adjusted socioeconomic model

*Description: Forest plot displaying rate ratios and 95% confidence intervals from the Poisson mixed-effects regression model including socioeconomic context, area of residence, sex, and their interactions.*
